# Knockout of ABC transporter gene *ABCA2* confers resistance to Bt toxin Cry2Ab in *Helicoverpa zea*

**DOI:** 10.1038/s41598-022-21061-2

**Published:** 2022-10-06

**Authors:** Jeffrey A. Fabrick, Chan C. Heu, Dannialle M. LeRoy, Ben A. DeGain, Alex J. Yelich, Gopalan C. Unnithan, Yidong Wu, Xianchun Li, Yves Carrière, Bruce E. Tabashnik

**Affiliations:** 1 USDA ARS, U.S. Arid Land Agricultural Research Center, 21881 N. Cardon Lane, Maricopa, AZ 85138 USA; 2 Department of Entomology, University of Arizona, Tucson, AZ 85721 USA; 3 College of Plant Protection, Nanjing Agricultural University, Nanjing, 210095 China

**Keywords:** Agricultural genetics, Molecular evolution, Agricultural genetics, Molecular biology, Functional genomics

## Abstract

Evolution of pest resistance reduces the benefits of widely cultivated genetically engineered crops that produce insecticidal proteins derived from *Bacillus thuringiensis* (Bt). Better understanding of the genetic basis of pest resistance to Bt crops is needed to monitor, manage, and counter resistance. Previous work shows that in several lepidopterans, resistance to Bt toxin Cry2Ab is associated with mutations in the gene encoding the ATP-binding cassette protein ABCA2. The results here show that mutations introduced by CRISPR/Cas9 gene editing in the *Helicoverpa zea* (corn earworm or bollworm) gene encoding ABCA2 (*HzABCA2*) can cause resistance to Cry2Ab. Disruptive mutations in *HzABCA2* facilitated the creation of two Cry2Ab-resistant strains. A multiple concentration bioassay with one of these strains revealed it had > 200-fold resistance to Cry2Ab relative to its parental susceptible strain. All Cry2Ab-resistant individuals tested had disruptive mutations in *HzABCA2*. We identified five disruptive mutations in *HzABCA2* gDNA. The most common mutation was a 4-bp deletion in the expected Cas9 guide RNA target site. The results here indicate that *HzABCA2* is a leading candidate for monitoring Cry2Ab resistance in field populations of *H. zea*.

## Introduction

Genetically engineered crops that produce insecticidal proteins from *Bacillus thuringiensis* (Bt) are useful for managing some economically important pests. Benefits of Bt crops include better pest control and decreased use of conventional insecticides, which enhances yields and farmer profits, and confers health and environmental benefits^1–6^. However, the evolution of resistance to Bt crops by pests decreases these benefits^7^. Understanding the genetic basis of resistance to Bt crops can improve the monitoring and management of resistance, as well strategies to counter pest resistance.

Some ATP-binding cassette (ABC) transporter proteins mediate Bt intoxication in insects^8–10^. Natural mutations in *ABCC2* are genetically linked with resistance to several Bt toxins in the Cry1 family (Cry1Ab, Cry1Ac, and/or Cry1Fa) in seven lepidopterans^11–16^. Likewise, resistance to Cry2Ab is linked with natural mutations in another ABC transporter gene, *ABCA2*, in *Helicoverpa armigera*^17^, *Helicoverpa punctigera*^17^, *Trichoplusia ni*^18^, and in lab- and field-selected populations of *Pectinophora gossypiella*^19,20^. Furthermore, knockout mutations in *ABCA2* generated in the laboratory using CRISPR/Cas9 or TALENs caused resistance to Cry2Ab in *H. armigera*^21^, *T. ni*^18^, *Bombyx mori*^22^, and *P. gossypiella*^23^. These results provide compelling evidence that ABCA2 plays an important role in the mode of action of Cry2Ab in these lepidopterans.

Here we tested the hypothesis that CRISPR/Cas9-mediated disruption of the gene encoding ABCA2 can cause resistance to Cry2Ab in *Helicoverpa zea* (also known as corn earworm and bollworm). This lepidopteran is a major pest of many crops in the New World, including corn, cotton, soybean, and sorghum^4,24^. It has evolved practical resistance to Bt corn and cotton producing Cry1 and Cry2A toxins throughout much of the southern United States^7,25–32^. Also, early signs of *H. zea* evolution of resistance to Vip3Aa produced by Bt crops have been reported^29,33^. Hence, strategies to delay or overcome the evolution of Bt resistance in *H. zea* are urgently needed. Although timely identification of genes causing Bt resistance remains challenging, new tools have spurred progress with *H. zea* and *H. armigera*^34–36^.

In this study, we discovered that introducing disruptive CRISPR/Cas9-mediated mutations in the *HzABCA2* gene in a susceptible strain caused resistance to Cry2Ab. We also used CRISPR/Cas9 to introduce disruptive mutations in the *HzTO* gene (also known as *tryptophan 2,3-oxygenase* or *vermilion*), which confirmed previous results showing this causes yellow eye color in *H. zea*^37^. All Cry2Ab-resistant individuals tested were found to harbor disruptive mutations in *HzABCA2* DNA in the single-guide RNA (sgRNA) target sites used for gene editing. These findings indicate that ABCA2 is important in toxicity of Cry2Ab to *H. zea*.

## Results

### In vitro Cas9 cleavage of *HzABCA2* PCR products with guide RNAs

We tested seven *HzABCA2* sgRNAs separately by combining the sgRNA/Cas9 ribonucleotide mixture for each sgRNA (#1–7) with gDNA amplified by PCR from *HzABCA2*. Cas9 cleavage of the *HzABCA2* amplicons in vitro occurred with sgRNA 7, but not sgRNAs 1–6 (Supplementary Fig. S1).

### Creation of Cry2Ab-resistant strains Hz-R2 and Yellow-R2

For our experimental design, we targeted *HzABCA2* to assess its role in Cry2Ab resistance and *HzTO* as a secondary and visible marker for gene editing. Of 177 embryos (G_0_) from the susceptible LAB-S strain injected with the ribonucleotide mixture containing *HzTO* sgRNA and *HzABCA2* sgRNA 7, 97% hatched and 73% pupated. To start the Hz-R2 strain, we pooled 55 G_0_ pupae (one cup with 10 males + 10 females, one cup with 10 males + 9 females, and one cup with 8 males + 8 females) and allowed the adults to eclose and mate. We established the Yellow-R2 strain by pairing two G_0_ adult females that had yellow eyes (Supplementary Fig. S2) with two Hz-R2 G_0_ males having mosaic green/yellow eyes and allowing them to propagate. We screened G_1_ neonates from both Hz-R2 and Yellow-R2 on diet containing the diagnostic concentration of 1 μg Cry2Ab per cm^2^ diet. Survival was 58% for Yellow-R2 (n = 224) and 93% for Hz-R2 (n = 192), suggesting that CRISPR/Cas9 efficiently introduced germline mutations in *HzABCA2* that caused resistance to Cry2Ab. Survival was 0% for LAB-S (n = 32), which is significantly lower than survival of the G_1_ larvae from either Hz-R2 or Yellow-R2 (Fisher’s exact test, P < 0.0001 for each comparison). In contrast, survival on control diet did not differ significantly between LAB-S (91%) and either Hz-R2 (88%) or Yellow-R2 (100%) (Fisher’s exact test, P > 0.5 in each comparison). We used the G_1_ survivors of exposure to Cry2Ab to continue the Yellow-R2 and Hz-R2 strains. In effect, this exposure constituted a single selection for resistance to Cry2Ab after which both strains were reared without exposure to the toxin.

**Figure 1:**
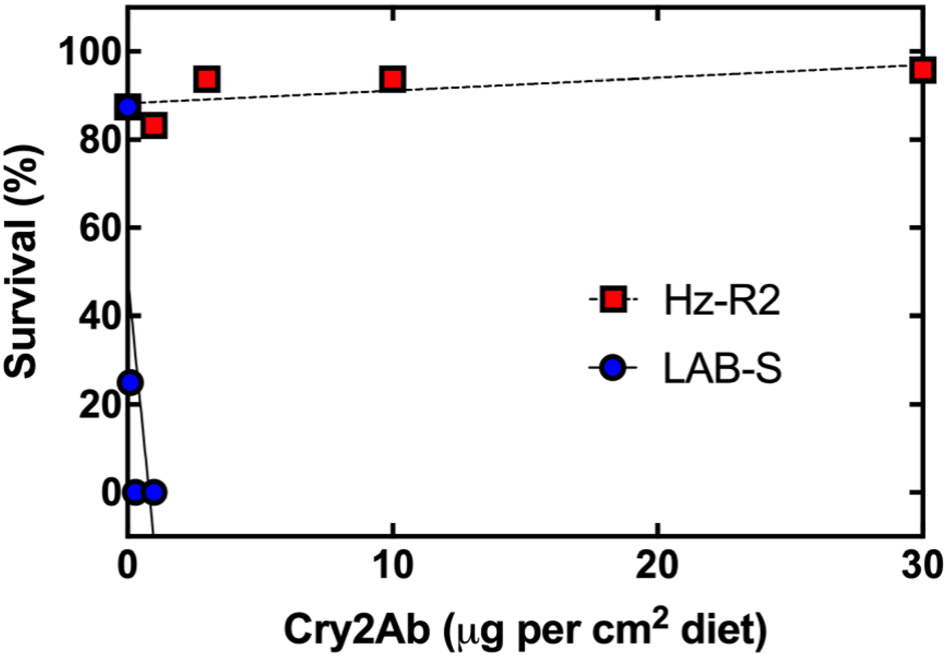
Susceptibility to Cry2Ab of CRISPR-edited strain Hz-R2 and its parent susceptible strain LAB-S of *H. zea*. Each point represents mean survival (n = 48 per point).

Analysis of the G_4_ larvae from Hz-R2 revealed they were highly resistant to Cry2Ab relative to their parent strain LAB-S (Fig. 1). Survival for Hz-R2 was 96% for larvae exposed to diet with 30 μg Cry2Ab per cm^2^ diet (the highest concentration tested). The concentration of Cry2Ab killing 50% of larvae (LC_50_ in μg Cry2Ab per cm^2^ diet) was > 30 for the G_4_ larvae from Hz-R2 and 0.14 (95% CI 0.10–0.19) for LAB-S, which yields a resistance ratio of > 217 for Hz-R2 relative to LAB-S.

To initiate Yellow-R2 containing the desired eye phenotype, we sorted G_2_ pupae by sex, selected adults with yellow eyes (14 males + 14 females) and allowed them to mate. After six generations of laboratory rearing without additional selection for yellow eyes, all 1,200 individuals examined from the G_7-9_ Yellow-R2 strain had yellow eyes (n = 400 per generation). This suggests that a HzTO mutation responsible for yellow eyes was likely fixed in the Yellow-R2 strain.

### Mutations in sgRNA target sites in *HzABCA2 and HzTO* gDNA from the Yellow-R2 and Hz-R2 strains

**Figure 2:**
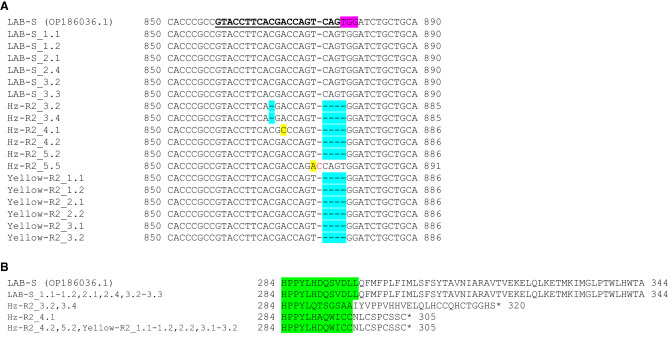
Mutations in *HzABCA2* gDNA and the resulting translated sequences from three Hz-R2 larvae and three Yellow-R2 that survived exposure to diet treated with 1 μg Cry2Ab per cm^2^ diet. (**A**) Partial *HzABCA2* genomic DNA fragments corresponding to *HzABCA2* sgRNA 7 were cloned and Sanger sequenced. Two clones from each of three Hz-R2 larvae and three Yelllow-R2 larvae (2 clones × 3 individuals) and two clones from three LAB-S moths were sequenced. *HzABCA2* sgRNA 7 sequence is bold and underlined. The PAM sequence is highlighted in pink. The DNA insertion is shown with red text, deletions are highlighted in blue, and substitutions are highlighted in yellow. Numbers indicate nucleotide position in the *HzABCA2* mRNA because only the exon section is shown. (**B**) Alignment of translated *HzABCA2* partial amino acid sequences from the Yellow-R2 and Hz-R2 G_1_ survivors on 1 μg Cry2Ab per cm^2^. Amino acids highlighted in green correspond to the gDNA Sanger sequenced for the corresponding *HzABCA2* sgRNA 7 target site. Stars indicate premature stop codons. Numbers indicate the position of the amino acid in the HzABCA2 protein sequence.

For *HzABCA2*, we sequenced two clones corresponding to exon 4 (containing *HzABCA2* sgRNA 7) from three Hz-R2 and Yellow-R2 G_1_ survivors reared on diet treated with 1 μg Cry2Ab per cm^2^ (total of 12 clones) and three LAB-S individuals on untreated diet (total of six clones; Fig. 2). All 12 clones from Hz-R2 and Yellow-R2 had mutations in the *HzABCA2* sgRNA 7 target site (Fig. 2A) which introduce premature stop codons (Fig. 2B; Supplementary Fig. S3). In total, we found five unique mutations in *HzABCA2* from Hz-R2 and Yellow-R2: a 1-bp deletion at base 867, a 4-bp deletion at position 875–878, an A869C substitution, an A874T substitution, and a 1-bp insertion after base 874. The most common mutation was the 4-bp deletion (bases 875-CAGT-878), which occurred in five of six Hz-R2 clones and all six Yellow-R2 clones. No mutations were found in exon 4 of *HzABCA2* in the six clones sequenced from LAB-S.

For *HzTO*, we sequenced two clones corresponding to exon 6 (the site of the *HzTO* sgRNA) from four Yellow-R2 G_1_ moths that had yellow eyes (total of 8 clones) and two clones from one LAB-S moth. Whereas both clones from LAB-S had the wild-type *HzTO* sequence, all eight clones from Yellow-R2 harbored disruptive mutations (Supplementary Fig. S4A). Overall, we found three different mutations in the *HzTO* gDNA from Yellow-R2, including a 2-bp insertion in one clone, a 7-bp deletion in five clones, and a 33-bp insertion in two clones (Supplementary Fig. S4A). All three CRISPR/Cas9-induced mutations disrupt the *HzTO* coding sequence with premature stop codons. The 7-bp deletion and the 2-bp insertion introduced a premature stop at the same codon position in *HzTO* (corresponding to bases 831 and 840, respectively) and the 33-bp insertion introduced a stop codon in the final 3 bp of the inserted sequence (Supplementary Figs. S4B, S5).

### Inheritance of resistance to Cry2Ab in strain Hz-R2

**Figure 3:**
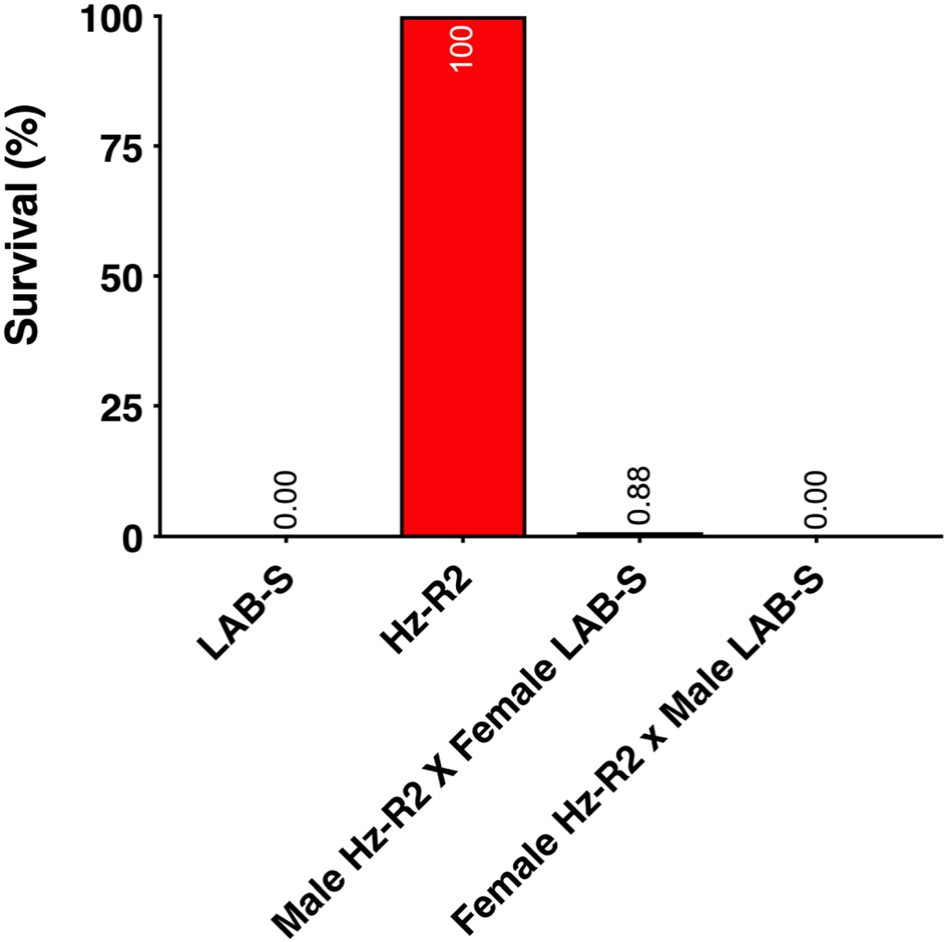
Survival of *H. zea* larvae from Hz-R2, its parent susceptible strain (LAB-S), and their F_1_ progeny from mass crosses. Mean corrected survival was determined using diet overlay bioassays with 1 μg Cry2Ab per cm^2^ diet for LAB-S, Hz-R2 (G_4_), and the two reciprocal crosses (n = 130 per bar).

To evaluate inheritance of resistance to Cry2Ab, we used diet overlay bioassays to test Hz-R2, LAB-S, and their F_1_ progeny resulting from mass crosses. Responses of the F_1_ progeny show that resistance to Cry2Ab in Hz-R2 was autosomal and recessive (Fig. 3). At the diagnostic concentration, survival was 0% for LAB-S, 100% for Hz-R2, and 0.4% for the F_1_ progeny (pooled from the two Hz-R2 X LAB-S reciprocal crosses).

The dominance parameter (*h*), which varies from 0 for completely recessive resistance to 1 for completely dominant resistance, was determined for the F_1_ progeny from pooled Hz-R2 X LAB-S mass crosses. The results yield *h* = 0.0041, indicating recessive inheritance of resistance for Hz-R2 at 1 μg Cry2Ab per cm^2^ diet.

## Discussion

The results here show that introduction of disruptive mutations in the *H. zea ABCA2* gene (*HzABCA2*) via CRISPR/Cas9 gene editing caused resistance to Cry2Ab. This finding is consistent with previous results from editing the homologous gene in *H. armigera, T. ni, B. mori*, and *P. gossypiella*^18,21–23^. Collectively these data indicate that ABCA2 is important for toxicity of Cry2Ab in at least five species representing three families of Lepidoptera (Noctuidae, Bombycidae, and Gelechiidae). Despite its evident functional importance, the precise role of this protein in the mode of action of Cry2Ab remains obscure^10^. Heckel (2021) has proposed that elucidating the three-dimensional structures of the ABC transporters will be critical for progress in understanding how these proteins facilitate pore formation by Bt toxins.

Here, we determined that the Hz-R2 strain of *H. zea*, which harbored CRISPR/Cas9-induced mutations in *HzABCA2*, was > 200-fold resistant to Cry2Ab compared to its susceptible parental strain LAB-S. Similarly, CRISPR/Cas9 knockout of *ABCA2* in two *H. armigera* lab strains from China revealed Cry2Ab resistance ratios of > 100 relative to the susceptible parental SCD strain^21^.

The Cry2Ab resistance in lab-selected strains of *H. armigera* and *H. punctigera* from Australia is linked with naturally occurring mutations in *ABCA2*^17^. One of the Cry2Ab-resistant strains of *H. armigera* from Australia called SP15 was generated from an F_2_ screen conducted in 2002^38^. Larval survival (adjusted for control mortality) at the highest concentration of Cry2Ab tested was 96% for Hz-R2 and 96% for SP15^38^. The highest concentration tested was 1.6 times higher for Hz-R2 than SP15 (30 versus 19 μg Cry2Ab per cm^2^ diet), which means the concentration killing 4% of larvae was higher for Hz-R2 than SP15. In the Australian study^38^, the mean LC_50_ (in μg Cry2Ab per cm^2^ diet) of two unrelated susceptible strains of *H. armigera* was 0.13, which is nearly identical to the LC_50_ of 0.14 for the susceptible LAB-S strain tested in this study. Thus, the resistance to Cry2Ab relative to conspecific susceptible strains was similar or slightly greater for the CRISPR-generated mutations in Hz-R2 than for the naturally occurring mutations in SP15. Consistent with the results for Hz-R2, resistance to Cry2Ab was recessive in strains of *H. armigera* from China and Australia as well as in *H. punctigera*^17,21,38^.

Whereas our study using CRISPR/Cas9 and related gene editing studies can rigorously test if mutations in specific genes confer resistance, they cannot determine if mutations in the genes evaluated actually occur in field-selected resistant populations of pests. Accordingly, a key question related to this study is the extent to which mutations disrupting ABCA2 contribute to field-evolved resistance to Cry2Ab. In *P. gossypiella* from India, mutations disrupting ABCA2 were associated with field-selected resistance to Cry2Ab^19,20^. In *H. armigera* and *H. punctigera* from Australia, mutations disrupting ABCA2 were associated with resistance to Cry2Ab in lab-selected strains derived from field populations^17^. However, these naturally occurring mutations affecting ABCA2 were not common in field populations of either species in Australia^17^, which remain susceptible to Cry2Ab^39^.

The results here showing that mutations disrupting ABCA2 can cause resistance to Cry2Ab in *H. zea* make it especially compelling to determine if such mutations are associated with the extensive field-evolved resistance of this pest to Cry2Ab in the southern United States^7,27–32^. In a single-pair family established from *H. zea* collected from corn producing Cry1A.105 + Cry2Ab in Maryland in 2019, resistance to these two Bt toxins was not associated with chromosome 17 where *HzABCA2* occurs^40^. More extensive screening of *H. zea* from field populations resistant to Cry2Ab is needed to determine if mutations disrupting ABCA2 are important in this widespread practical resistance.

## Methods

### Insects

We bought *H. zea* eggs from Benzon Research (Carlisle, PA) in July 2021. We maintained this laboratory strain without exposure to Bt toxins and refer to it as LAB-S because of its susceptibility to Cry2Ab and other Bt toxins^27,28,31,41^. Larvae were reared on Southland diet (Southland Products, Inc., Lake Village, AR). All rearing and diet bioassays were done at 28 °C under 20–40% humidity and 14 h light:10 h dark. Moths were reared in separate incubators from larvae and had access to a 10% sugar water solution for feeding and cheese cloth for oviposition^41^.

### Bt toxin

We used Cry2A.127, a variant of Cry2Ab protoxin that was prepared, purified, and solubilized as described previously^42^ and provided by Corteva Agriscience. Cry2A.127 is 98.6% identical with Cry2Ab1 and Cry2Ab2 (9 substitutions out of 633 amino acids) and is referred to here as Cry2Ab.

### Design and synthesis of single guide RNA (sgRNA)

We designed sgRNAs targeting two genes: (1) *HzABCA2* encoding the ATP-binding cassette protein ABCA2 (from the *H. zea* genome^36^); and (2) *vermilion* (also known as *tryptophan 2,3-oxygenase* or *HzTO*) MG976796.1. The latter gene was previously shown to result in yellow mutant eye color in CRISPR/Cas9 mutant knockouts of *H. zea*^37^. One sgRNA located in *HzTO* exon 6 and seven sgRNAs located within 5' exons of *HzABCA2* were made (*HzABCA2* sgRNAs 1 and 2 in exon 1; sgRNAs 3 and 4 in exon 2; sgRNA 5 in exon 3; and sgRNAs 6 and 7 in exon 4) (Supplementary Table S1). Each sgRNA was adjacent to a corresponding “NGG” PAM site and had no off-target sites based on CRISPOR alignment with *H. armigera ABCA2* and *TO*. Each selected sgRNA target sequence was further checked for potential off-target sites by BLAST searching of the GenBank non-redundant database. sgRNA DNA templates were synthesized as gBlock DNA (Integrated DNA Technologies, Coralville, Iowa) that contained the T7 RNA polymerase binding site, a 20-bp gene-specific target sequence, and the 80-bp common stem-loop tracrRNA sequence. DNA templates were used to synthesize sgRNA using the HiScribe T7 High Yield RNA synthesis Kit (New England Biolabs, Ipswich, MA). Transcribed sgRNAs were treated with DNase I for 20 min at 37 °C and purified using RNAClean XP (Thermo Fisher Scientific) following the manufacturer’s protocol.

### In vitro Cas9 cleavage with guide RNAs

To test if each sgRNA complexed with Cas9 was able to cut PCR-amplified *HzABCA2* gDNA in vitro, we used the Guide-it sgRNA Screening Kit (Takara Bio, Mountain View, CA) as previously published^23^. We first extracted gDNA from LAB-S 3^rd^ instar larvae using the Gentra Puregene Tissue Kit (Qiagen, Hilden, Germany), which served as DNA template for PCR. Primer pairs 1HzABCA2-5 + 2HzABCA2-3, 3HzABCA2-5 + 4HzABCA2-3, and 5HzABCA2-5 + 6HzABCA2-3 (Supplementary Table S2) were used to amplify PCR products corresponding to exon 1, exon 2, and exons 3–4, respectively. Each sgRNA was diluted to 50 ng μL^−1^ in RNase-free water and 1 μL was mixed with 250 ng of Guide-it Cas9 nuclease and incubated at 37 °C for 5 min. Fifty ng of each gDNA PCR template was combined with Cas9 Reaction Buffer, bovine serum albumin, RNase-free water, and the appropriate Cas9/sgRNA mixture. These were incubated at 37 °C for 1 h. Reactions were terminated (80 °C for 5 min) and aliquots of each digestion reaction and corresponding negative controls were analyzed by 1.5% agarose gel electrophoresis stained with SYBR Safe DNA Gel Stain (Thermo Fisher Scientific).

### Embryo microinjection

sgRNA targeting *HzABCA2* and *HzTO* were singly complexed with the Alt-R *Streptococcus pyogenes* HiFi Cas9 nuclease V3 (Integrated DNA Technologies), at 50 ng µL^−1^ of sgRNA to 100 ng µL^−1^ of Cas9, then incubated at room temperature for 15 min to generate the Cas9-ribonucleoprotein (RNP) complex. The two RNP complexes were combined to create a mixture of *HzABCA2* and *HzTO* RNP, then the mixture was placed on ice and immediately used for injections.

Embryos were collected from LAB-S by placing microscope glass coverslips (24 X 40 mm, Corning Inc., Corning, NY) on top of screened lids for several cages containing 5 male and 5 female adults for 45 min. Adult *H. zea* females naturally affix eggs to the coverslips and no additional manipulation of the embryos was needed. An IM-300 microinjector (Narishige International USA, Amityville, NY) equipped with an Olympus IMT-2 inverted microscope (Olympus Corporation, Center Valley, PA) was used to inject newly laid eggs (less than 1 h old) with approximately 100–200 picoliters of the *HzTO* + *HzABCA2* RNP solution. Quartz needles (Sutter Instrument Co, Novato, CA) were beveled using a Model EG-44 micropipette grinder (Narishige) at a 30° angle and a rotor speed of ~ 1800 rpm or 90% of the maximum speed. Needles were backfilled with 3 µL of the RNP mix using Eppendorf Microloader pipette tips. Following injection, the coverslips containing embryos (n = 177 total injected embryos) were placed into 100 X 15 mm Petri dishes containing 1% agarose and held at 28 °C until G_0_ neonates emerged.

Newly emerged G_0_ neonates were transferred to individual 30 mL translucent polystyrene cups containing 10 mL of Southland diet and reared at 28 °C (14:10 L:D) to pupation. Pupae (n = 125) were sexed and transferred to individual 30 mL cups until adult eclosion. Two G_0_ adult females showing full yellow eyes were allowed to mate with two G_0_ males showing mosaic eyes to generate Yellow-R2 G_1_ neonates used in diet selection bioassays (below). The remainder of the surviving G_0_ adults (28 males and 27 females) were placed into cages with 8–10 males and 8–10 females and used to generate Hz-R2 G_1_ neonates used for selection (below).

### Creation of the Cry2Ab-resistant strains Hz-R2 and Yellow-R2

We tested G_1_ larvae from the unselected Yellow-R2 and Hz-R2 strains for susceptibility to Cry2Ab using our standard 7-d diet overlay bioassays^41^. We tested one neonate per well in bioassay trays (BIO-BA-128, Pitman, NJ) on diet treated with 1 μg Cry2Ab per cm^2^ diet (n = 192 for Hz-R2 and n = 224 for Yellow-R2) or on control diet (n = 16) treated with 40 µl of 0.1% Triton X-100 (no Cry2Ab). Trays were covered with Pull N' Peel covers (BIO-CU-16, Pitman, NJ). Survivors (≥ 4th instar larvae at 7 days) on Cry2Ab-treated diet were used to further propagate the Hz-R2 and Yellow-R2 strains. We calculated adjusted survival (%) as the survival (%) on diet containing 1 μg Cry2Ab per cm^2^ divided by survival (%) on untreated diet. We used a two-tailed Fisher's exact test (http://www.graphpad.com/quickcalcs/contingency1/) to determine if a significant difference occurred between LAB-S and the G_1_ of each CRISPR-edited strain in the proportion of live larvae on treated diet and on untreated diet.

### Cas9-induced mutations in gDNA target regions

To determine if G_1_ larvae of Yellow-R2 and Hz-R2 that survived on diet surface treated with 1 μg Cry2Ab per cm^2^ harbored mutations corresponding to sgRNA target sites, we amplified, cloned, and DNA sequenced the relevant *HzABCA2* and *HzTO* gDNA corresponding to sgRNA target sites. We extracted gDNA separately from LAB-S, Yellow-R2, and Hz-R2 larvae using the DNeasy Blood and Tissue kit (Qiagen, Hilden, Germany). Phusion Green High-Fidelity DNA Polymerase (Thermo Fisher Scientific) was used with oligonucleotide primers (Supplementary Table S2) to amplify specific regions corresponding to both *HzABCA2* and *HzTO* sgRNA target sites. PCR conditions were 98 °C for 1 min for 1 cycle; 98 °C for 5 s, 48 °C for 5 s, and 72 °C for 10 s for 35 cycles; 72 °C for 1 min; and hold at 16 °C. PCR amplicons were analyzed on 1.5% agarose gels and stained with SYBR Safe DNA Gel Stain (Thermo Fisher Scientific). DNA bands were excised, cloned into the pJET1.2 vector (Thermo Fisher Scientific), and transformed into One Shot TOP10 Chemically Competent *E. coli* (Thermo Fisher Scientific). Purified plasmid DNA was Sanger sequenced by Retrogen (San Diego, CA). Multiple sequence alignments were performed using MUSCLE^43^. The full-length *HzABCA2* sequence from LAB-S is deposited in the GenBank public database (Accession number OP186036.1).

### Susceptibility to Cry2Ab of Hz-R2 and LAB-S

We used diet overlay bioassays^41^ to determine the concentration of Cry2Ab necessary to kill 50% of larvae (LC_50_) for Hz-R2 and LAB-S. Newly emerged neonates (n = 48 larvae per concentration) were placed into separate wells of bioassay trays. For LAB-S these contained 0, 0.1, 0.3, or 1 μg Cry2Ab per cm^2^ diet, and for Hz-R2 (G_4_) they contained 0, 1, 3, 10, and 30 μg Cry2Ab per cm^2^ diet. Trays were held at 28 °C (14 h light:10 h dark). After 7 days, we scored live larvae that were third or higher instars as survivors. We adjusted mortality for control mortality using Abbott’s correction and calculated LC_50_ using R (v 3.6.3)^44^ and the publicly available script https://github.com/JuanSilva89/Probit-analysis/commit/2eaaff05da0f89294788bd0bed564e1bf257acf2. The resistance ratio was calculated by dividing the LC_50_ for Hz-R2 by the LC_50_ for LAB-S.

### Inheritance of resistance to Cry2Ab in strain Hz-R2: maternal effects, sex linkage, and dominance

We evaluated the mode of inheritance of resistance to Cry2Ab for Hz-R2 by testing F_1_ neonates from reciprocal Hz-R2 X LAB-S crosses in diet overlay bioassays on 1 μg Cry2Ab per cm^2^ diet (n = 130 neonates per cross) and control diet (n = 32 neonates per cross). Survivors (≥ 4th instar larvae) were determined after 7 days of diet exposure and adjusted % survival was calculated by dividing survival on treated diet by survival on diet without Cry2Ab. We evaluated the dominance parameter *h*, which varies from 0 for completely recessive resistance to 1 for completely dominant resistance^45^. We calculated *h* for Hz-R2 as: *h* = (F_1_ survival − S survival)/(R survival − S survival), where the S is LAB-S, R is Hz-R2, and F_1_ is larvae resulting from reciprocal crosses between Hz-R2 and LAB-S.

## Supplementary Information

Supplementary Information.

## Data Availability

The full-length HzABCA2 cDNA sequence (OP186036.1) generated during the current study is available in the NCBI GenBank repository (https://www.ncbi.nlm.nih.gov/genbank/).
