# Supplementary material for: Knockout of ABC transporter gene ABCA2 confers resistance to Bt toxin Cry2Ab in Helicoverpa zea
Source: Sci Rep. 2022 Oct 6;12:16706. doi: 10.1038/s41598-022-21061-2 (PMC9537329; doi:10.1038/s41598-022-21061-2)
Supplement: Supplementary file 1 — Supplementary Information. [file 41598_2022_21061_MOESM1_ESM.pdf]

SUPPLEMENTARY INFORMATION

Knockout of ABC transporter gene *ABCA2* confers resistance to Bt toxin Cry2Ab in *Helicoverpa zea*

Jeffrey A. Fabrick[1,*], Chan C. Heu[1], Dannialle M. LeRoy[1], Ben A. DeGain[2], Alex J. Yelich[2], Gopalan C. Unnithan[2], Yidong Wu[3], Xianchun Li[2], Yves Carrière[2], and Bruce E. Tabashnik[2]

[1] USDA ARS, U.S. Arid Land Agricultural Research Center, Maricopa, AZ 85138 USA
[2] Department of Entomology, University of Arizona, Tucson, AZ 85721 USA
[3] College of Plant Protection, Nanjing Agricultural University, Nanjing 210095, China

[*] Corresponding author:

Jeffrey A. Fabrick
USDA ARS, U.S. Arid Land Agricultural Research Center
21881 N. Cardon Lane
Maricopa, AZ 85138, USA
Phone: 520-316-6335
Email: jeff.fabrick@usda.gov

SUPPLEMENTARY INFORMATION includes:

Supplementary Tables S1-S2

Supplementary Figures S1-S5

Supplementary Table S1. *HzABCA2* and *HzTO* Single Guide RNAs (sgRNAs).

sgRNA	Sequence (5' to 3')[1]	Position[2]	Exon	Direction
HzABCA2-1	ACATAAGCTAATACGACTCACTATA**GAAAGTTCTTCCACATGAGC**GTTTTAGAGCTAGAAATAGCAAGTTAAA ATAAGGCTAGTCCGTTATCAACTTGAAAAAGTGGCACCGAGTCGGTGCTTTT	48-67	1	Antisense
HzABCA2-2	ACATAAGCTAATACGACTCACTATA**AAGGCGGTATCTGCCACCGA**GTTTTAGAGCTAGAAATAGCAAGTTAAA ATAAGGCTAGTCCGTTATCAACTTGAAAAAGTGGCACCGAGTCGGTGCTTTT	147-166	1	Antisense
HzABCA2-3	ACATAAGCTAATACGACTCACTATA**ATCTAACACTGGACTTGCCG**GTTTTAGAGCTAGAAATAGCAAGTTAAA ATAAGGCTAGTCCGTTATCAACTTGAAAAAGTGGCACCGAGTCGGTGCTTTT	275-294	2	Antisense
HzABCA2-4	ACATAAGCTAATACGACTCACTATA**TTTTATTGATAACTTACCAC**GTTTTAGAGCTAGAAATAGCAAGTTAAA ATAAGGCTAGTCCGTTATCAACTTGAAAAAGTGGCACCGAGTCGGTGCTTTT	357-376	2	Sense
HzABCA2-5	ACATAAGCTAATACGACTCACTATA**TTTCCTCACTCGTGGGAAGG**GTTTTAGAGCTAGAAATAGCAAGTTAAA ATAAGGCTAGTCCGTTATCAACTTGAAAAAGTGGCACCGAGTCGGTGCTTTT	703-722	3	Sense
HzABCA2-6	ACATAAGCTAATACGACTCACTATA**TGCCCTTGATACCAGCTCCA**GTTTTAGAGCTAGAAATAGCAAGTTAAA ATAAGGCTAGTCCGTTATCAACTTGAAAAAGTGGCACCGAGTCGGTGCTTTT	782-801	4	Antisense
HzABCA2-7	ACATAAGCTAATACGACTCACTATA**GTACCTTCACGACCAGTCAG**GTTTTAGAGCTAGAAATAGCAAGTTAAA ATAAGGCTAGTCCGTTATCAACTTGAAAAAGTGGCACCGAGTCGGTGCTTTT	858-877	4	Sense
HzTO-1	ACATAAGCTAATACGACTCACTATA**AGTTGAACCCGTGAGTCGTG**GTTTTAGAGCTAGAAATAGCAAGTTAAA ATAAGGCTAGTCCGTTATCAACTTGAAAAAGTGGCACCGAGTCGGTGCTTTT	603-622	6	Antisense

[1] Sequence corresponding to the T7 RNA polymerase binding site (dashed underline); target gene-specific sgRNA sequence (bold); common stem-loop TracrRNA sequence (underline).
[2] Sequence position within *HzABCA2* (OP186036.1) or *HzTO* (MG976796.1) cDNA, respectively.

Supplementary Table S2. Nucleotide primers used to amplify, genotype, and/or DNA sequence *HzABCA2* and *HzTO*.

Primer	Sequence	Direction	Application
1HzABCA2-5	5' - ATGAGATTAGAAACGAGGCACG - 3'	Sense	*HzABCA2* exon 1 primer for amplification of gDNA for Cas9 *in vitro* screen and PCR amplicon DNA sequencing
2HzABCA2-3	5' - AGGATATTGGTAGAGTAGTTGAGTGTG - 3'	Antisense	*HzABCA2* exon 1 primer for amplification of gDNA for Cas9 *in vitro* screen and PCR amplicon DNA sequencing
3HzABCA2-5	5' - TTTTGCCATGAATATGGAAGAA - 3'	Sense	*HzABCA2* exon 2 primer for amplification of gDNA for Cas9 *in vitro* screen and PCR amplicon DNA sequencing
4HzABCA2-3	5' - CATATAGTTTATCGTCAAACTCGACG - 3'	Antisense	*HzABCA2* exon 2 primer for amplification of gDNA for Cas9 *in vitro* screen
5HzABCA2-5	5' - GAGCAGAACAGTTGTCAAATAATTT - 3'	Sense	*HzABCA2* exon 3 primer for amplification of gDNA for Cas9 *in vitro* screen and PCR amplicon DNA sequencing
6HzABCA2-3	5' - CTTTAATTGCAATTCCTTCTCAA - 3'	Antisense	*HzABCA2* exon 4 primer for amplification of gDNA for Cas9 *in vitro* screen and PCR amplicon DNA sequencing
1HzTO-5	5' - GCCCGATTCGAAAACTTCTT - 3'	Sense	*HzTO* PCR amplicon DNA sequencing
2HzTO-3	5' - ATGACGGCAGATAGAAGAATGG - 3'	Antisense	*HzTO* PCR amplicon DNA sequencing
3HzTO-5	5' - GTTGACAGCAGCCTGGAACT - 3'	Sense	*HzTO* PCR amplicon DNA sequencing
4HzTO-3	5' - TGGCCTTTCCTCAACTTCAA - 3'	Antisense	*HzTO* PCR amplicon DNA sequencing

Supplementary Figure S1. *In vitro* screen of sgRNAs by Cas9 cleavage of *HzABCA2* PCR gDNA amplicons. A) Cas9/sgRNA ribonucleotide mixtures incubated with *HzABCA2* gDNA PCR products were separated by 1.5% agarose gel electrophoresis. Genomic DNA PCR products (exon 1 = 1HzABCA2-5+2HzABCA2-3; exon 2 = 3HzABCA2-5+4HzABCA2-3; exons 3-4 = 5HzABCA2-5+6HzABCA2-3) were incubated with or without *HzABCA2* sgRNAs (1-7). Negative (-) and positive (+) symbols indicate the absence or presence of either sgRNA and/or Cas9, respectively. The red arrow indicates primary band resulting from Cas9 cleavage in presence of sgRNA 7. Lanes labeled "M" contain 1 kb Plus DNA Ladder (Thermo Fisher Scientific). B) Unprocessed agarose gel showing same *in vitro* screen of sgRNAs by Cas9 cleavage of *HzABCA2* PCR gDNA amplicons as above in A.

A

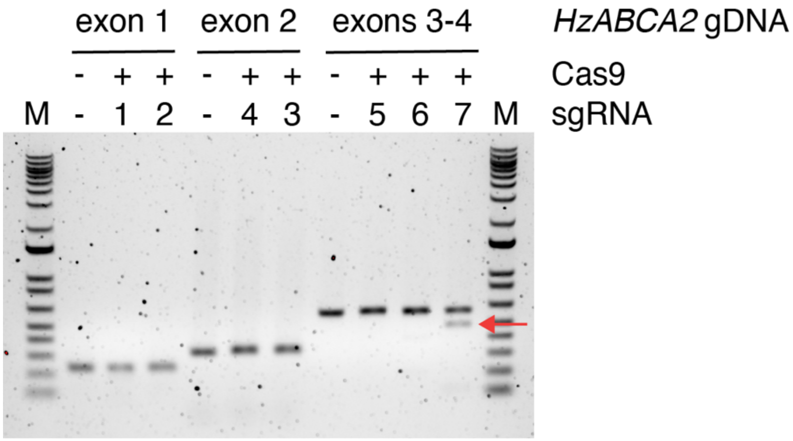

B

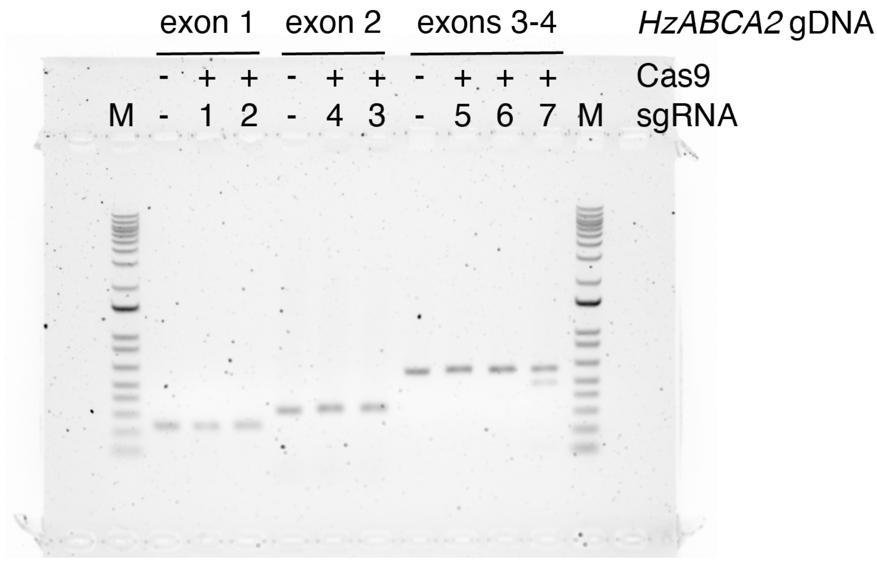

Supplementary Figure S2. Eye pigmentation for moths from the wild-type LAB-S strain and the CRISPR-edited Yellow-R2 strain of *H. zea*. A) Ventral view and B) lateral view of a LAB-S moth with wild-type green eyes under light adapted conditions (light on for 2.5 hours). C) Ventral view and D) lateral view of a Yellow-R2 moth with yellow eyes under light adapted conditions. E) Ventral view and F) lateral view of a LAB-S moth with wild-type black eyes under dark adapted conditions (light off for 10 hours). G) Ventral view and H) lateral view of a Yellow-R2 moth with yellow eyes under dark adapted conditions. Bar = 1 mm.

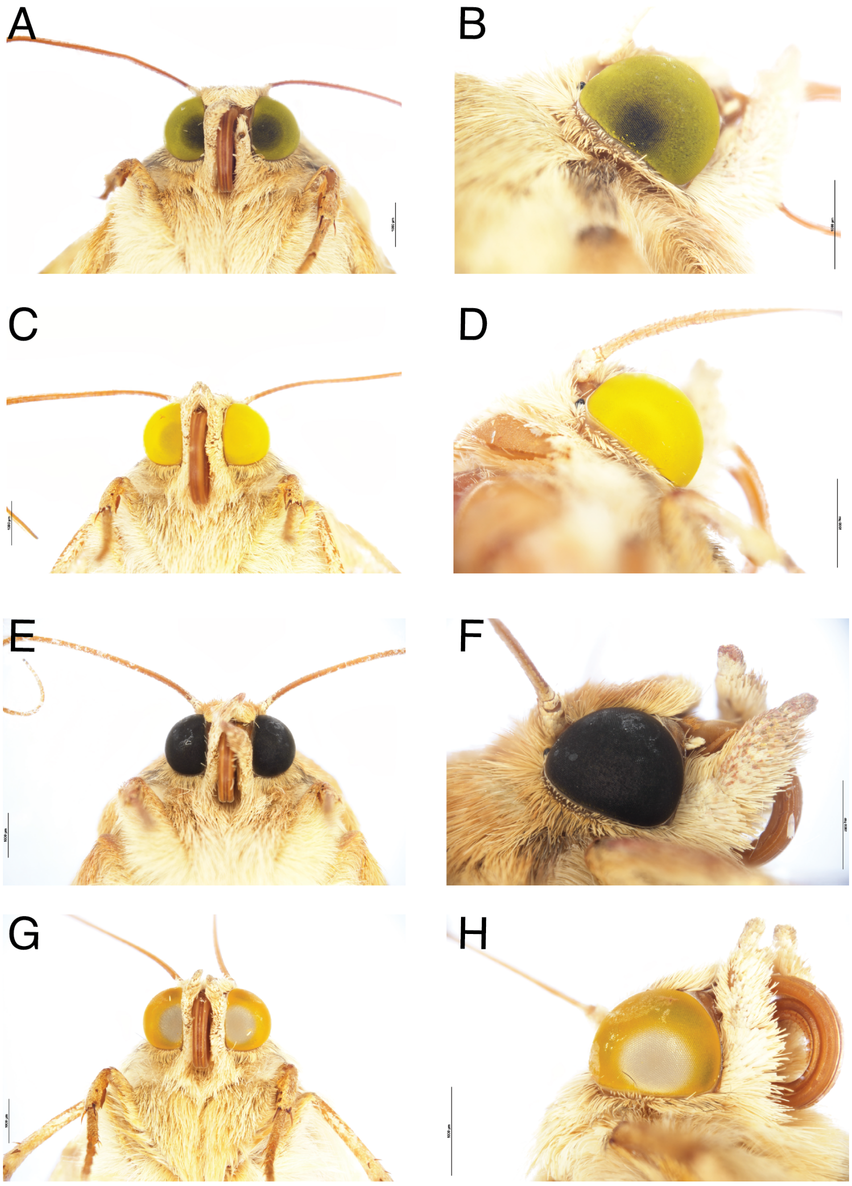

Supplementary Figure S3. Alignment of predicted *HzABCA2* translated amino acid sequences from Yellow-R2 and Hz-R2 G_1 survivors on 1 µg Cry2Ab per cm^2 with gDNA mutations corresponding to the *HzABCA2* sgRNA 7 target site. Translated *HzABCA2* sequences predicted from six survivors on Cry2Ab diet from Yellow-R2 and Hz-R2 were aligned with *HzABCA2* from LAB-S using Clustal MUSCLE (https://www.ebi.ac.uk/Tools/msa/muscle/). Names of translated sequences correspond to strain name, individual number, and clone number (e.g., Hz-R2_4.1). Green highlighted sequence shows each of the sequenced regions corresponding to the *HzABCA2* sgRNA 7 target site. Stars show amino acids conserved in all the sequences.

```
Hz-R2_3.2,3.4                                 MRLETRHASAATKFRLLMWKNFLQQWRHRLQTVVELLLPVVTMALVLILRWQIPPYQIDT
LAB-S_1.1-1.2,2.1,2.4,3.2-3.3                 MRLETRHASAATKFRLLMWKNFLQQWRHRLQTVVELLLPVVTMALVLILRWQIPPYQIDT
Hz-R2_4.1                                     MRLETRHASAATKFRLLMWKNFLQQWRHRLQTVVELLLPVVTMALVLILRWQIPPYQIDT
Hz-R2_4.2,5.2,Yellow-R2_1.1-1.2,2.2,3.1-3.2   MRLETRHASAATKFRLLMWKNFLQQWRHRLQTVVELLLPVVTMALVLILRWQIPPYQIDT
                                              ************************************************************

Hz-R2_3.2,3.4                                 LTYPALPAHTLNYSTNILFAMNMEELSIAYSPASPVLDDVMRTAVINLLTANMKDLIPIF
LAB-S_1.1-1.2,2.1,2.4,3.2-3.3                 LTYPALPAHTLNYSTNILFAMNMEELSIAYSPASPVLDDVMRTAVINLLTANMKDLIPIF
Hz-R2_4.1                                     LTYPALPAHTLNYSTNILFAMNMEELSIAYSPASPVLDDVMRTAVINLLTANMKDLIPIF
Hz-R2_4.2,5.2,Yellow-R2_1.1-1.2,2.2,3.1-3.2   LTYPALPAHTLNYSTNILFAMNMEELSIAYSPASPVLDDVMRTAVINLLTANMKDLIPIF
                                              ************************************************************

Hz-R2_3.2,3.4                                 IDNLPPGIANITFPPDMNLNTSAIEEFVKSRIRVVPYNSSYEIRGIYVDEETTRSIIAAV
LAB-S_1.1-1.2,2.1,2.4,3.2-3.3                 IDNLPPGIANITFPPDMNLNTSAIEEFVKSRIRVVPYNSSYEIRGIYVDEETTRSIIAAV
Hz-R2_4.1                                     IDNLPPGIANITFPPDMNLNTSAIEEFVKSRIRVVPYNSSYEIRGIYVDEETTRSIIAAV
Hz-R2_4.2,5.2,Yellow-R2_1.1-1.2,2.2,3.1-3.2   IDNLPPGIANITFPPDMNLNTSAIEEFVKSRIRVVPYNSSYEIRGIYVDEETTRSIIAAV
                                              ************************************************************

Hz-R2_3.2,3.4                                 EFDDKLYGAEQLSNNLSYSLRFPERPRLNSFFQTGGRTWRSDGVFPVFEVPGPRFPHSWE
LAB-S_1.1-1.2,2.1,2.4,3.2-3.3                 EFDDKLYGAEQLSNNLSYSLRFPERPRLNSFFQTGGRTWRSDGVFPVFEVPGPRFPHSWE
Hz-R2_4.1                                     EFDDKLYGAEQLSNNLSYSLRFPERPRLNSFFQTGGRTWRSDGVFPVFEVPGPRFPHSWE
Hz-R2_4.2,5.2,Yellow-R2_1.1-1.2,2.2,3.1-3.2   EFDDKLYGAEQLSNNLSYSLRFPERPRLNSFFQTGGRTWRSDGVFPVFEVPGPRFPHSWE
                                              ************************************************************

Hz-R2_3.2,3.4                                 GGNDPGYVNEMFVALQQVISMELVSRATGLDLKSFRVNIQRYPHPPYLQTSGSAAI----
LAB-S_1.1-1.2,2.1,2.4,3.2-3.3                 GGNDPGYVNEMFVALQQVISMELVSRATGLDLKSFRVNIQRYPHPPYLHDQSVDLLQFMF
Hz-R2_4.1                                     GGNDPGYVNEMFVALQQVISMELVSRATGLDLKSFRVNIQRYPHPPYLHAQWICC-----
Hz-R2_4.2,5.2,Yellow-R2_1.1-1.2,2.2,3.1-3.2   GGNDPGYVNEMFVALQQVISMELVSRATGLDLKSFRVNIQRYPHPPYLHDQWICC-----
                                              ************************************************: .

Hz-R2_3.2,3.4                                 ------------------------------------------------------------
LAB-S_1.1-1.2,2.1,2.4,3.2-3.3                 PLFIMLSFSYTAVNIARAVTVEKELQLKETMKIMGLPTWLHWTAWFVKQFIYLSITAVLL
Hz-R2_4.1                                     ------------------------------------------------------------
Hz-R2_4.2,5.2,Yellow-R2_1.1-1.2,2.2,3.1-3.2   ------------------------------------------------------------

Hz-R2_3.2,3.4                                 ------------------------------------------------------------
LAB-S_1.1-1.2,2.1,2.4,3.2-3.3                 VVLLKVNWFTNDDGFSEYAVFTNTPWTVLLFFLILYLSCAIFFSFMVSSIFSKGSTAALF
Hz-R2_4.1                                     ------------------------------------------------------------
Hz-R2_4.2,5.2,Yellow-R2_1.1-1.2,2.2,3.1-3.2   ------------------------------------------------------------

Hz-R2_3.2,3.4                                 ------------------------------------------------------------
LAB-S_1.1-1.2,2.1,2.4,3.2-3.3                 MAVAWFLTYIPAFLLAMDINMSTAVQVITCFSINSAMSYGFQLMLAKESTGGLQWGDFMT
Hz-R2_4.1                                     ------------------------------------------------------------
Hz-R2_4.2,5.2,Yellow-R2_1.1-1.2,2.2,3.1-3.2   ------------------------------------------------------------

Hz-R2_3.2,3.4                                 ------------------------------------------------------------
LAB-S_1.1-1.2,2.1,2.4,3.2-3.3                 SPGTDTTRFVFGHVVIMLVVDCLIYMLITLYLEQVLPGPFGTPKPWYFPFQLQFWFPNYK
Hz-R2_4.1                                     ------------------------------------------------------------
Hz-R2_4.2,5.2,Yellow-R2_1.1-1.2,2.2,3.1-3.2   ------------------------------------------------------------

Hz-R2_3.2,3.4                                 ------------------------------------------------------------
LAB-S_1.1-1.2,2.1,2.4,3.2-3.3                 SKDAGLIFENDNSEFDDIIKEKDPTDHEVGVKMQNLTKIFGNNIAVNNLSLNIYDDQITV
Hz-R2_4.1                                     ----------------------------------NL------------------------
Hz-R2_4.2,5.2,Yellow-R2_1.1-1.2,2.2,3.1-3.2   ----------------------------------NL------------------------
```

```
Hz-R2_3.2,3.4                                           ------------------------------------------------------------
LAB-S_1.1-1.2,2.1,2.4,3.2-3.3                           LLGHNGAGKSTTISMLTGNLKVTRGTVNVAGYDMTSQSSAARSHIGLCPQHNILFNELTV
Hz-R2_4.1                                               ------------------------------------------------------------
Hz-R2_4.2,5.2,Yellow-R2_1.1-1.2,2.2,3.1-3.2             ------------------------------------------------------------

Hz-R2_3.2,3.4                                           ------------------------------------------------------------
LAB-S_1.1-1.2,2.1,2.4,3.2-3.3                           KEHLEFFARLKGFKGKELYEEIDSLIEKLELQEKRDYPSKGLSGGQKRRLCVGIALSGAA
Hz-R2_4.1                                               ------------------------------------------------------------
Hz-R2_4.2,5.2,Yellow-R2_1.1-1.2,2.2,3.1-3.2             ------------------------------------------------------------

Hz-R2_3.2,3.4                                           ------------------------------------------------------------
LAB-S_1.1-1.2,2.1,2.4,3.2-3.3                           RVVLLDEPTSGMDPSSRRALWELLQKEKKGRSMILTTHFMDEADILGDRVAIMANGRLQC
Hz-R2_4.1                                               ------------------------------------------------------------
Hz-R2_4.2,5.2,Yellow-R2_1.1-1.2,2.2,3.1-3.2             ------------------------------------------------------------

Hz-R2_3.2,3.4                                           ------------------------------------------------------------
LAB-S_1.1-1.2,2.1,2.4,3.2-3.3                           VGSPYFLKRHYGVGYTLVIVKDTDFDFVKCSVLINSYIPGTIVKEDRGTEITYNLVNDYS
Hz-R2_4.1                                               ------------------------------------------------------------
Hz-R2_4.2,5.2,Yellow-R2_1.1-1.2,2.2,3.1-3.2             ------------------------------------------------------------

Hz-R2_3.2,3.4                                           ------------------------------------------------------------
LAB-S_1.1-1.2,2.1,2.4,3.2-3.3                           HVFEEMLNDLERNIDNIKFKNYGLVATTLEDVFMSVGADLSPINSESDDAITTTTDSTID
Hz-R2_4.1                                               ------------------------------------------------------------
Hz-R2_4.2,5.2,Yellow-R2_1.1-1.2,2.2,3.1-3.2             ------------------------------------------------------------

Hz-R2_3.2,3.4                                           ----------------------------------------------YVPPVHHV------
LAB-S_1.1-1.2,2.1,2.4,3.2-3.3                           DILKQEIDSSLEELDKDESNVTGLRLFGQQVLAVWMKQWLVLIRSPWVMVLQFLAPVVLI
Hz-R2_4.1                                               ------------------------------------------------------------
Hz-R2_4.2,5.2,Yellow-R2_1.1-1.2,2.2,3.1-3.2             ------------------------------------------------------------

Hz-R2_3.2,3.4                                           ------------------------------------------------------------
LAB-S_1.1-1.2,2.1,2.4,3.2-3.3                           NSTLGVLRYVMSLSPTIRTRWLSLEEGYTESETLLSFNGSVASSVGALAAQAYQSLFANS
Hz-R2_4.1                                               ------------------------------------------------------------
Hz-R2_4.2,5.2,Yellow-R2_1.1-1.2,2.2,3.1-3.2             ------------------------------------------------------------

Hz-R2_3.2,3.4                                           ------------------------------------------------------------
LAB-S_1.1-1.2,2.1,2.4,3.2-3.3                           GVMDMEINAIGSQPIEEYYLNRTSDPVVMGSLRHRLLIGSTFDDNSATAWFSNFGYHDVA
Hz-R2_4.1                                               ------------------------------------------------------------
Hz-R2_4.2,5.2,Yellow-R2_1.1-1.2,2.2,3.1-3.2             ------------------------------------------------------------

Hz-R2_3.2,3.4                                           ------------------------------------------------------------
LAB-S_1.1-1.2,2.1,2.4,3.2-3.3                           TSLAAIHSAILRSKNSDAVLNVYNHPLEASYIDQSDVQTMIAMLSMQLSSGIGSSVSIVS
Hz-R2_4.1                                               ------------------------------------------------------------
Hz-R2_4.2,5.2,Yellow-R2_1.1-1.2,2.2,3.1-3.2             ------------------------------------------------------------

Hz-R2_3.2,3.4                                           ------------------------------------------------------------
LAB-S_1.1-1.2,2.1,2.4,3.2-3.3                           AVFIMFYIKERMSGAKLLQNAAGVAPSVLWGGAAIFNWFWFLITCVSIVISCVAFDVIGL
Hz-R2_4.1                                               ------------------------------------------------------------
Hz-R2_4.2,5.2,Yellow-R2_1.1-1.2,2.2,3.1-3.2             ------------------------------------------------------------

Hz-R2_3.2,3.4                                           ------------------------------------------------------------
LAB-S_1.1-1.2,2.1,2.4,3.2-3.3                           SNVHELGRMFLCVMVYGAAMLPLVYLLSLKFKGPAVGFVGFYFLNVLFGMMGAQVVEALS
Hz-R2_4.1                                               ------------------------------------------------------------
Hz-R2_4.2,5.2,Yellow-R2_1.1-1.2,2.2,3.1-3.2             ------------------------------------------------------------

Hz-R2_3.2,3.4                                           ------------------------------------------------------------
LAB-S_1.1-1.2,2.1,2.4,3.2-3.3                           SPMLDTEQAAHILDYLLQFYPLYSLVTSIRFLNQVGLREYTCLQGCEYLQAVYPNLECSM
Hz-R2_4.1                                               ------------------------------------------------------------
Hz-R2_4.2,5.2,Yellow-R2_1.1-1.2,2.2,3.1-3.2             ------------------------------------------------------------

Hz-R2_3.2,3.4                                           ----ELQLHCCQH----------------CTGGHS-------------------------
LAB-S_1.1-1.2,2.1,2.4,3.2-3.3                           ASMCEFHSNCCVRENPYFDWEEPGVLRYLLSMCFSCLIFWLLLMTIEYRVVQKVFTFKKT
Hz-R2_4.1                                               -----------------------------CSPCSSC------------------------
Hz-R2_4.2,5.2,Yellow-R2_1.1-1.2,2.2,3.1-3.2             -----------------------------CSPCSSC------------------------
                                                                                    :   *
```

```
Hz-R2_3.2,3.4                                  ------------------------------------------------------------
LAB-S_1.1-1.2,2.1,2.4,3.2-3.3                  PPPIDESTLDEDVMTEARRARQVPPTRRSDHALLAHDLSKYYGKHLAVDQVSFSVNDGEC
Hz-R2_4.1                                      ------------------------------------------------------------
Hz-R2_4.2,5.2,Yellow-R2_1.1-1.2,2.2,3.1-3.2    ------------------------------------------------------------

Hz-R2_3.2,3.4                                  ------------------------------------------------------------
LAB-S_1.1-1.2,2.1,2.4,3.2-3.3                  FGLLGVNGAGKTTTFKMLMGDESISSGEAYVSGHSVQRNLDRVHENIGYCPQFDALFGEL
Hz-R2_4.1                                      ------------------------------------------------------------
Hz-R2_4.2,5.2,Yellow-R2_1.1-1.2,2.2,3.1-3.2    ------------------------------------------------------------

Hz-R2_3.2,3.4                                  ------------------------------------------------------------
LAB-S_1.1-1.2,2.1,2.4,3.2-3.3                  TGRQTLHMFALMRGLRLRTAAPSAETLAHALGFFKHLDKRVHQYSGGTKRKLNTAIAFMG
Hz-R2_4.1                                      ------------------------------------------------------------
Hz-R2_4.2,5.2,Yellow-R2_1.1-1.2,2.2,3.1-3.2    ------------------------------------------------------------

Hz-R2_3.2,3.4                                  ------------------------------------------------------------
LAB-S_1.1-1.2,2.1,2.4,3.2-3.3                  RTRLVFVDEPTTGVDPAAKRHVWRATRGVQRAGRGVVLTSHSMEECEALCSRLTIMVNGR
Hz-R2_4.1                                      ------------------------------------------------------------
Hz-R2_4.2,5.2,Yellow-R2_1.1-1.2,2.2,3.1-3.2    ------------------------------------------------------------

Hz-R2_3.2,3.4                                  ------------------------------------------------------------
LAB-S_1.1-1.2,2.1,2.4,3.2-3.3                  FQCLGTPQHLKNKFSQGFTLIIKMKTDDSDSDTQSVNSTTSVVDSVKLYVSGNFESPKIM
Hz-R2_4.1                                      ------------------------------------------------------------
Hz-R2_4.2,5.2,Yellow-R2_1.1-1.2,2.2,3.1-3.2    ------------------------------------------------------------

Hz-R2_3.2,3.4                                  ------------------------------------------------------------
LAB-S_1.1-1.2,2.1,2.4,3.2-3.3                  EEYHGLLTYYLPDRSMAWSRMFGIMERAKQILQIEDYSISQTTLEQIFLQFTKYQREEGT
Hz-R2_4.1                                      ------------------------------------------------------------
Hz-R2_4.2,5.2,Yellow-R2_1.1-1.2,2.2,3.1-3.2    ------------------------------------------------------------

Hz-R2_3.2,3.4                                  --
LAB-S_1.1-1.2,2.1,2.4,3.2-3.3                  TL
Hz-R2_4.1                                      --
Hz-R2_4.2,5.2,Yellow-R2_1.1-1.2,2.2,3.1-3.2    --
```

Supplementary Figure S4. Mutations in *HzTO* gDNA and the resulting translated sequences from four Yellow-R2 moths with yellow eyes. A) Partial *HzTO* genomic DNA fragments corresponding with the *HzTO* sgRNA target site were cloned and Sanger sequenced. Two clones from each of four Yelllow-R2 moths with yellow eye phenotype (2 clones x 4 individuals) and two clones from one LAB-S moth were sequenced. The *HzTO* sgRNA sequence is in bold and underlined. The PAM sequence is highlighted in pink. DNA insertions are shown with red text and deletions are highlighted in blue. B) Alignment of translated *HzTO* partial amino acid sequences from the four Yellow-R2 moths. Amino acids highlighted in green correspond to the gDNA Sanger sequenced for the corresponding *HzTO* sgRNA target site in exon 6. Stars indicate premature stop codons.

A

```
MG976796.1       585 GGAGCGTACACCCGGCCTCACG---------------------------------ACTCACGGGTTCAACTTCTGGGGC 630
LAB-S_1          585 GGAGCGTACACCCGGCCTCACG---------------------------------ACTCACGGGTTCAACTTCTGGGGC 630
LAB-S_2          585 GGAGCGTACACCCGGCCTCACG---------------------------------ACTCACGGGTTCAACTTCTGGGGC 630
Yellow-R2_1.1    585 GGAGCGTACACCCGGCCTCACGGG-------------------------------ACTCACGGGTTCAACTTCTGGGGC 632
Yellow-R2_1.2    585 GGAGCGTACACCCGGC-----------------------------------------CTCACGGGTTCAACTTCTGGGGC 623
Yellow-R2_2.1    585 GGAGCGTACACCCGGC-----------------------------------------CTCACGGGTTCAACTTCTGGGGC 623
Yellow-R2_2.2    585 GGAGCGTACACCCGGC-----------------------------------------CTCACGGGTTCAACTTCTGGGGC 623
Yellow-R2_3.1    585 GGAGCGTACACCCGGC-----------------------------------------CTCACGGGTTCAACTTCTGGGGC 623
Yellow-R2_3.2    585 GGAGCGTACACCCGGC-----------------------------------------CTCACGGGTTCAACTTCTGGGGC 623
Yellow-R2_4.1    585 GGAGCGTACACCCGGCCTCACGGGATGTAGGGAGGGTAGTAGGGTCGGGATGTAGACTCACGGGTTCAACTTCTGGGGC 663
Yellow-R2_4.2    585 GGAGCGTACACCCGGCCTCACGGGATGTAGGGAGGGTAGTAGGGTCGGGATGTAGACTCACGGGTTCAACTTCTGGGGC 663
```

B

```
MG976796.1                          196 ERTPGLT-----THGFNFWGKFQAAVNKLIKDDIDAASRETNETVRRHRLQDAENRREIYRSIFDPAVHDALRSRGERRLSHKAL 275
LAB-S_1,2                           196 ERTPGLT-----THGFNFWGKFQAAVNKLIKDDIDAASRETNETVRRHRLQDAENRREIYRSIFDPAVHDALRSRGERRLSHKAL 275
Yellow-R2_1.1                       196 ERTPGLTGLTGSTSGASSRLLSTNSLRMILMLLLVKQTRRFDATGCRTRRTGARSTAPSSTLPSTTPSGPGENEDYPTRHYKAL* 280
Yellow-R2_1.2,2.1-2.2,3.1-3.2       196 ERTPGLT---GSTSGASSRLLSTNSLRMILMLLLVKQTRRFDATGCRTRRTGARSTAPSSTLPSTTPSGPGENEDYPTRHYKAL* 277
Yellow-R2_4.1-4.2                   196 ERTPGLT---GCREGSRVGM* 213
```

Supplementary Figure S5. Alignment of predicted *HzTO* translated amino acid sequences from Yellow-R2 G_1 survivors on 1 µg Cry2Ab per cm^2 with gDNA mutations corresponding to the sgRNA target site. Translated *HzTO* sequences predicted from four Yellow-R2 G_1 moths each having the yellow eye phenotype and two clones from a single control LAB-S moth were aligned with *HzTO* (MG976796.1) using MUSCLE (https://www.ebi.ac.uk/Tools/msa/muscle/). Names for Yellow-R2 translated sequences include individual number and clone number (e.g., Yellow-R2_1.1). Green highlighted sequence shows each of the sequenced region corresponding to the *HzTO* sgRNA target site. Stars show amino acids conserved in all the sequences.

```
MG976796.1                      MACPMRSALDESMAQEGDCLGNEAGMLYGEYLMLDKLLSAQRMLSAESSKPVHDEHLFIV
Yellow-R2_1.1                   MACPMRSALDESMAQEGDCLGNEAGMLYGEYLMLDKLLSAQRMLSAESSKPVHDEHLFIV
Yellow-R2_1.2,2.1-2,3.1-2       MACPMRSALDESMAQEGDCLGNEAGMLYGEYLMLDKLLSAQRMLSAESSKPVHDEHLFIV
Yellow-R2_4.1-2                 MACPMRSALDESMAQEGDCLGNEAGMLYGEYLMLDKLLSAQRMLSAESSKPVHDEHLFIV
                                ************************************************************

MG976796.1                      THQAYELWFKQIIFEVDSVRALLDVEGLDESHTMEILKRLNRIVLILKLLVDQVMILETM
Yellow-R2_1.1                   THQAYELWFKQIIFEVDSVRALLDVEGLDESHTMEILKRLNRIVLILKLLVDQVMILETM
Yellow-R2_1.2,2.1-2,3.1-2       THQAYELWFKQIIFEVDSVRALLDVEGLDESHTMEILKRLNRIVLILKLLVDQVMILETM
Yellow-R2_4.1-2                 THQAYELWFKQIIFEVDSVRALLDVEGLDESHTMEILKRLNRIVLILKLLVDQVMILETM
                                ************************************************************

MG976796.1                      TPLDFMDFRNYLRPASGFQSLQFRLLENKLGLKQALRVKYNQSYQTVFGDDPEAMDALQK
Yellow-R2_1.1                   TPLDFMDFRNYLRPASGFQSLQFRLLENKLGLKQALRVKYNQSYQTVFGDDPEAMDALQK
Yellow-R2_1.2,2.1-2,3.1-2       TPLDFMDFRNYLRPASGFQSLQFRLLENKLGLKQALRVKYNQSYQTVFGDDPEAMDALQK
Yellow-R2_4.1-2                 TPLDFMDFRNYLRPASGFQSLQFRLLENKLGLKQALRVKYNQSYQTVFGDDPEAMDALQK
                                ************************************************************

MG976796.1                      SEQEPALLALIERWLERTPGLTTHGFNFWGKFQAAVNKLIKDDIDAASRETNETVRRHRL
Yellow-R2_1.1                   SEQEPALLALIERWLERTPGLT----GLTGSTSGASSRL---------------------
Yellow-R2_1.2,2.1-2,3.1-2       SEQEPALLALIERWLERTPGLT-------GSTSGASSRL---------------------
Yellow-R2_4.1-2                 SEQEPALLALIERWLERTPGLT-------GCREGS-------------------------
                                **********************       *  ..:

MG976796.1                      QDAENRREIYRSIFDPAVHDALRSRGERRLSHKALQGAIMITFYRDEPRFSQPHQLLTLL
Yellow-R2_1.1                   -----------------------------LSTNSLRMILMLLLVKQTRRFDATG------
Yellow-R2_1.2,2.1-2,3.1-2       -----------------------------LSTNSLRMILMLLLVKQTRRFDATG------
Yellow-R2_4.1-2                 ------------------------------------------------------------

MG976796.1                      MDIDSLITKWRYNHVIMVQRMIGSQQLGTGGSSGYQYLRSTLSDRYKVFLDLFNLSTFLL
Yellow-R2_1.1                   ---------------------CRTRRTGARSTAPSSTLPST-------------------
Yellow-R2_1.2,2.1-2,3.1-2       ---------------------CRTRRTGARSTAPSSTLPST-------------------
Yellow-R2_4.1-2                 -------------------------RVGM-------------------------------
                                                         . *

MG976796.1                      PRSLIPPLDDGMKRSLNLTWGDSVKENGQNGDNAQNGDNAQNGIESSL
Yellow-R2_1.1                   ----------------------TPSGPGENEDYPTRHYKAL-------
Yellow-R2_1.2,2.1-2,3.1-2       ----------------------TPSGPGENEDYPTRHYKAL-------
Yellow-R2_4.1-2                 ------------------------------------------------
```
